# A Double-Humanized Mouse Model for Studying Host Gut Microbiome–Immune Interactions in Gulf War Illness

**DOI:** 10.3390/ijms25116093

**Published:** 2024-05-31

**Authors:** Dipro Bose, Punnag Saha, Subhajit Roy, Ayushi Trivedi, Madhura More, Nancy Klimas, Ashok Tuteja, Saurabh Chatterjee

**Affiliations:** 1 Environmental Health and Disease Laboratory, Department of Environmental and Occupational Health, Program in Public Health, Susan and Henry Samueli College of Health Sciences, University of California, Irvine, CA 92697, USA; diprob@uci.edu (D.B.); punnags@uci.edu (P.S.); subhajr@uci.edu (S.R.); aktrived@uci.edu (A.T.); mpmore@uci.edu (M.M.); 2 Institute for Neuro-Immune Medicine, Nova Southeastern University, Fort Lauderdale, FL 33328, USA; nklimas@nova.edu; 3 Division of Gastroenterology, University of Utah School of Medicine, Salt Lake City, UT 84132, USA; ashok.tuteja@hsc.utah.edu; 4 Division of Infectious Disease, School of Medicine, University of California, Irvine, CA 92697, USA; 5 VA Research and Development, VA Long Beach Health Care, Long Beach, CA 90822, USA

**Keywords:** humanized mice, NSG, bacteriome, IL-6, TNF R-1, gut–immune axis

## Abstract

Unraveling the multisymptomatic Gulf War Illness (GWI) pathology and finding an effective cure have eluded researchers for decades. The chronic symptom persistence and limitations for studying the etiologies in mouse models that differ significantly from those in humans pose challenges for drug discovery and finding effective therapeutic regimens. The GWI exposome differs significantly in the study cohorts, and the above makes it difficult to recreate a model closely resembling the GWI symptom pathology. We have used a double engraftment strategy for reconstituting a human immune system coupled with human microbiome transfer to create a humanized-mouse model for GWI. Using whole-genome shotgun sequencing and blood immune cytokine enzyme linked immunosorbent assay (ELISA), we show that our double humanized mice treated with Gulf War (GW) chemicals show significantly altered gut microbiomes, similar to those reported in a Veteran cohort of GWI. The results also showed similar cytokine profiles, such as increased levels of IL-1β, IL-6, and TNF R-1, in the double humanized model, as found previously in a human cohort. Further, a novel GWI Veteran fecal microbiota transfer was used to create a second alternative model that closely resembled the microbiome and immune-system-associated pathology of a GWI Veteran. A GWI Veteran microbiota transplant in humanized mice showed a human microbiome reconstitution and a systemic inflammatory pathology, as reflected by increases in interleukins 1β, 6, 8 (IL-1β, IL-6, IL-8), tumor necrosis factor receptor 1 (TNF R-1), and endotoxemia. In conclusion, though preliminary, we report a novel in vivo model with a human microbiome reconstitution and an engrafted human immune phenotype that may help to better understand gut–immune interactions in GWI.

## 1. Introduction

Significant research has been conducted, including clinical and preclinical studies, for the past 30 years on Gulf War Illness (GWI), advancing our understanding of complex multisymptomatic pathologies. Studies have reported the prevalence of GWI symptoms to be significantly higher among the Veterans who were deployed in the 1990–91 Gulf War (GW) compared to the non-deployed population [1]. It is important to note that a section of aging GW Veterans, in the present day, continue to experience the persistence of GWI symptoms [2].

GWI has been reported to be the result of the exposure to multiple chemical and environmental toxicants by Veterans during the war [3]. Several research groups, including ours, have attempted to administer two or more such reported toxicants as a mixture in preclinical murine models to understand the pathophysiology and exposome profile of GWI. However, most studies have used experimental mouse or rat strains to design their respective chronic or sub-chronic GWI models [4].

Recent approaches have focused on substituting experimental murine models with humanized mouse models. The advantage of the humanized mouse model lies in its ability to possess human immune cells, gut microbiome, specific tissues, and tumor growth [5]. Humanized mouse models have proven to be advantageous in studying the altered immune functions in autoimmune, metabolic, and infectious diseases [6,7]. Among the different strains of humanized mice that are used, CD34+ hu-NSG strain and BLT-TKO strain are the most commonly used. CD34+ hu-NSGs are non-obese diabetic (NOD) mice, where the gene coding for the γ chain of the IL-2 receptor is deleted, preventing cytokine signaling, along with a *scid* mutation in the *Prkdc* or DNA repair protein. They do not have any B- or T-cells or functional natural killer cells. The humanization of the immune system is conducted after the initial myeloablation through radiation, followed by injection with human CD34+ hematopoietic stem cells [8,9]. The approach for generating TKO-BLT-humanized mice with C57BL/6 backgrounds decreased its effectiveness because of the issue of graft rejection, as human CD47 cannot be recognized by mice signal regulatory protein α (SIRPα) expressed in C57BL/6 mice [10]. To overcome this, triple-knockout (TKO) mice with deletions of *Rag1*, *IL-2γc*, and *CD47* genes were used. Further engraftment with human CD34+ hematopoietic stem cells and human fetal tissues (bone marrow, liver, thymus, or BLT) led to the development of TKO-BLT-humanized mice, which were immunologically stable to transplantations [11]. Further modification of existing CD34+ hu-NSG- and TKO-BLT-humanized mouse models by engrafting with a human gut microbiome via fecal microbiota transfer (FMT) has led to the development of a “double humanized mouse model” [12]. This model has the advantage for being more translatable for mimicking the human-gut microbiome-induced host immune response as opposed to immune responses due to an altered murine microbiome, which are different [13].

GWI pathology has been identified to be associated with an altered gut microbiome due to GW chemical exposures [14,15,16,17]. We have observed in our previous studies that the diversity or richness of the gut microbiome, as measured by α-diversity, has been significantly altered in GWI Veterans and in preclinical mouse models [14,15,16]. Present-day GWI Veterans belong to the elderly population between 50 and 60 years old. Studies have reported that in the elderly population, the α-diversity is decreased because of exposure to nosocomial infections upon multiple hospital visits, sepsis, antibiotic prescriptions, and diet [18]. A lower α-diversity index, like the Chao 1 index, was associated with mortality among elderly patients [19]. α-Diversity could be used for its predictive value, especially as an indicator for the onset of neurodegeneration, which is majorly observed among the GWI Veteran cohort [20,21]. Hence, we have analyzed the α-diversity along with studying the inter-group gut bacterial variation through β-diversity analysis, which can help to identify signature gut bacterial profiles for designing future therapeutic approaches. Also, it will be more advantageous to utilize a double humanized mouse model to study the GWI pathology, as the immunological response could be more translatable to the clinical data available from GWI Veterans. In the present study, we have used CD34+ hu-NSG (NOD.Cg-*Prkdc*^scid^ *Il2rg*^tm1Wjl^/SzJ), mostly termed as NSG^TM^ mouse strains, which were engrafted with human hematopoietic stem cells (hu-CD34+). The CD34+ hu-NSG mice were further engrafted with a human gut microbiome from a healthy human subject after antibiotic-induced gut depletion via FMT, following the protocol reported by Daharsh et al., with modifications [12]. We have studied the gut microbiome and systemic immune responses after administering GW-representative chemicals in CD34+ hu-NSG mice with an established human gut microbiome. Further, we report the altered gut microbiome and immunological signature post-engrafting the CD34+ hu-NSG mice with a GWI Veteran’s microbiome by performing FMT using a stool sample from a GWI Veteran.

## 2. Results

### 2.1. Establishment of Healthy Human Gut Bacteriome in CD34+ hu-NSG Mice

**Figure 1:**
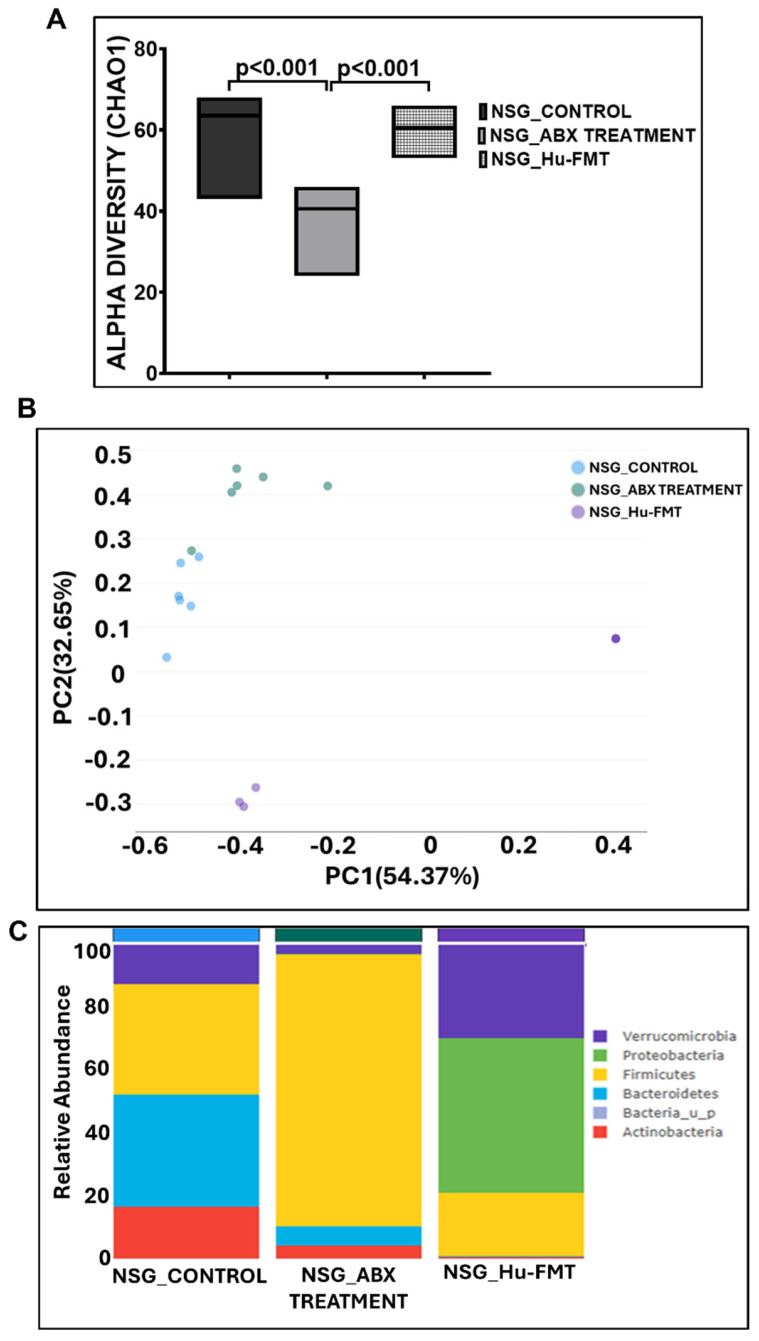
Establishment of human gut bacteria in NSG-CD34+ mice. (**A**) Box plots showing α-diversities (Chao 1) of gut bacteriome in NSG_Control group (mice administered with the vehicle), NSG_ABX Treatment (mice administered with antibiotic cocktail via oral gavage for 12 days), and NSG_Hu-FMT (mice administered with human fecal microbiota transfer after gut bacteriome depletion with antibiotic cocktail). (**B**) β-Diversity analysis (Bray–Curtis) of NSG_Control, NSG_ABX Treatment, and NSG_Hu-FMT groups. (**C**) Stacked bar representation of relative abundance of gut bacteriome at the phylum level in NSG_Control, NSG_ABX Treatment, and NSG_Hu-FMT groups; *p* < 0.05 was considered as statistically significant.

**Figure 2:**
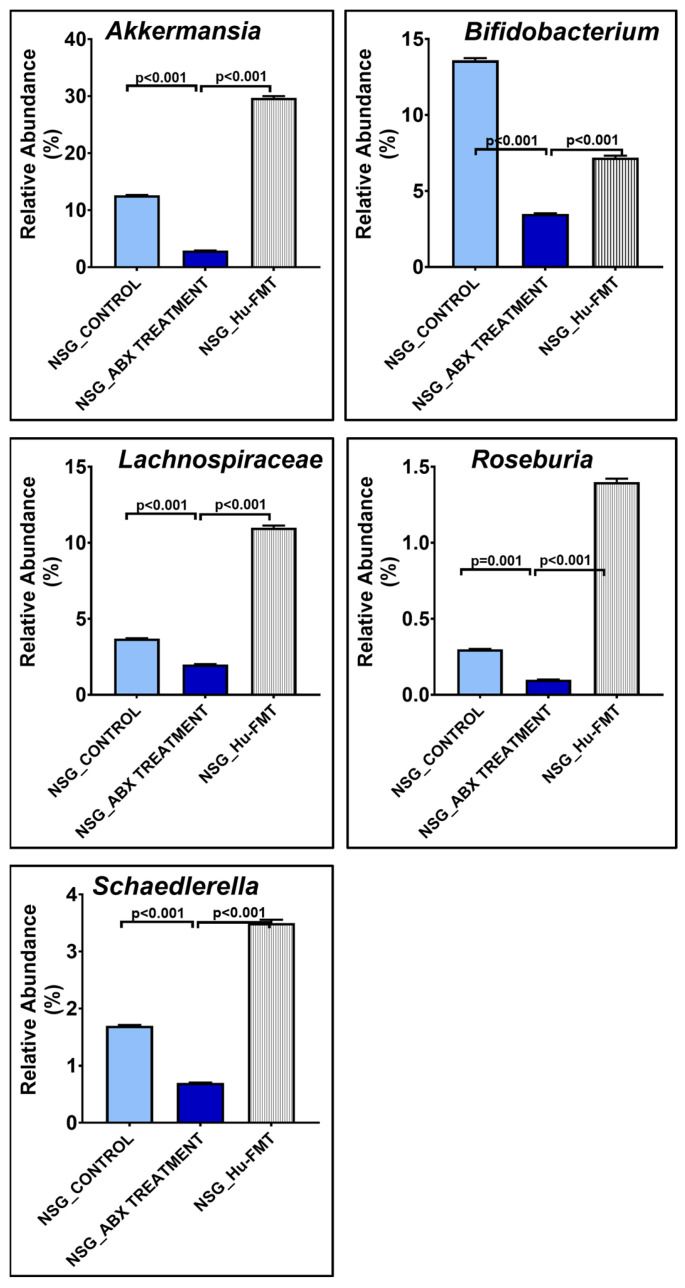
Bar graph representation of relative abundances of gut bacteriome at the genus level in NSG_Control, NSG_ABX Treatment, and NSG_Hu-FMT groups; *p* < 0.05 was considered as statistically significant.

**Figure 3:**
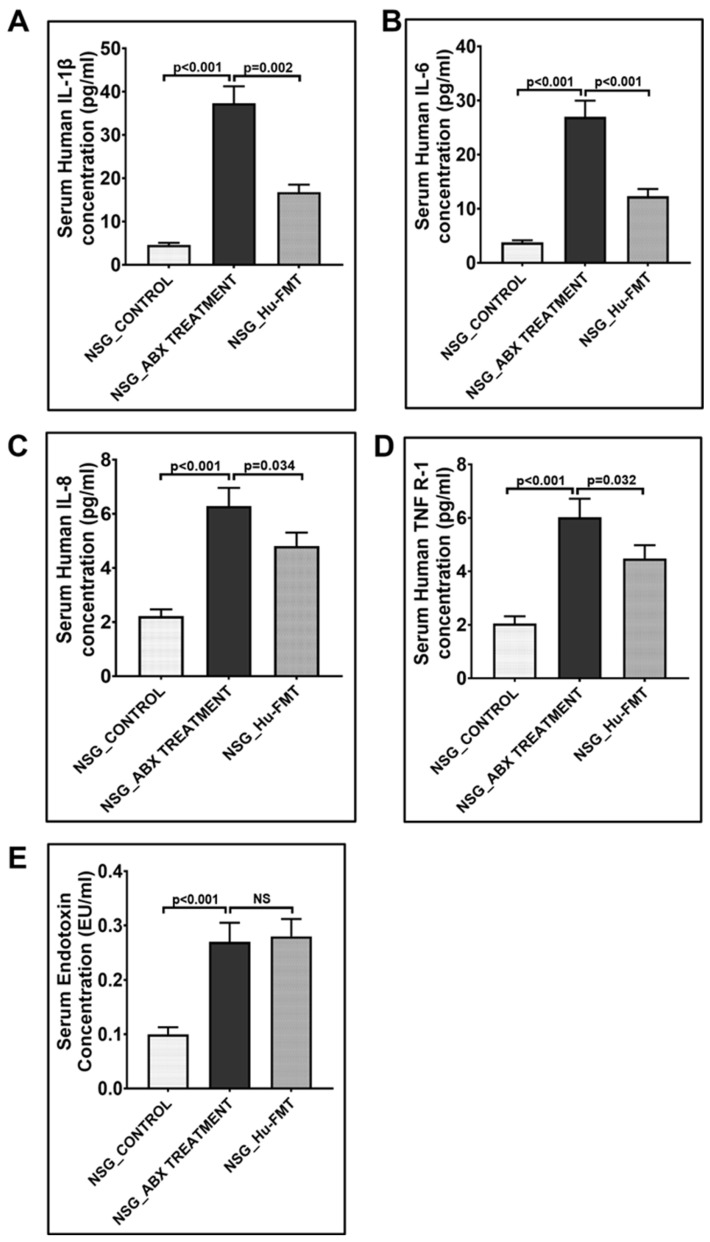
Altered expressions of systemic proinflammation biomarkers during establishment of human gut bacteria in NSG-CD34+ mice. Bar graph representation of systemic cytokines (**A**) IL-1β, (**B**) IL-6, (**C**) IL-8, and (**D**) TNF R-1 in NSG_Control, NSG_ABX Treatment, and NSG_Hu-FMT groups. (**E**) Bar graph representation of serum endotoxemia, measured by LAL Assay in NSG_Control, NSG_ABX Treatment, and NSG_Hu-FMT groups; *p* < 0.05 was considered as statistically significant. NS denotes non-significant change.

We wanted to establish the gut bacteriome of a healthy human donor in the CD34+ hu-NSG^TM^ mouse strain. Gut depletion was performed by administering an antibiotic cocktail via oral gavage for 12 days, followed by FMT with a healthy human fecal sample for 2 days. To study the efficacy of the antibiotic-cocktail-induced gut depletion and the subsequent human gut bacteriome establishment, we performed a whole-genome sequencing study using fecal samples from the three groups. The results showed that the α-diversity (Chao 1) of the gut bacteriome was significantly decreased in the NSG_ABX Treatment group compared to the NSG_Control group (*p* < 0.001) (Figure 1A). The administration of the human FMT after the initial gut depletion by antibiotic treatment in the NSG_Hu-FMT group resulted in a significant increase in the α-diversity (Chao 1) compared to the NSG_ABX Treatment group, which indicated the establishment of human gut bacteria (*p* < 0.001) (Figure 1A). The Bray–Curtis β-diversity analysis showed significant differences in the gut bacteriome profile between the NSG_Control and NSG_ABX Treatment groups (*p* < 0.002) and between the NSG_ABX Treatment and NSG_Hu-FMT groups (*p* < 0.005) (Figure 1B). At the phylum level, an increase in the relative abundance of *Firmicutes* and a decrease in *Verrucomicrobia* were observed in the NSG_ABX Treatment group as compared to the NSG_Control group (Figure 1C). Moreover, human FMT treatment increased the relative abundance of *Verrucomicrobia* and decreased *Firmicutes* in the NSG_Hu-FMT group compared to the NSG_ABX Treatment group (Figure 1C). Further investigating the colonized bacterial population in the CD34+ hu-NSG mouse strain by comparing it with the bacterial profile of the healthy human donor at the genus level (Supplementary Materials Table S1), we observed that the relative abundances of *Bifidobacterium* and *Roseburia* were significantly increased in NSG_Hu-FMT groups compared to the NSG_ABX Treatment group (Figure 2). We also observed significant increases in the relative abundances of beneficial gut commensal bacteria *Akkermansia*, *Lachnospiraceae*, and *Schaedlerella* in NSG_Hu-FMT groups compared to the NSG_ABX Treatment group (Figure 2). Further, we wanted to study the expression of the systemic inflammation in the three groups by performing serum enzyme-linked immunosorbent assays (ELISAs). We observed significantly increased expressions of human proinflammatory cytokines IL-1β, IL-6, and IL-8 and the human soluble tumor necrosis factor receptor 1 (TNF R-1) at the serum level in the NSG_ABX Treatment group compared to the NSG_Control group, which was significantly decreased in the NSG_Hu-FMT group (Figure 3A–D). There were no statistically significant differences in the endotoxin levels between the NSG_ABX Treatment and NSG_Hu-FMT groups (Figure 3E). These results suggested that the gut bacteriome depletion and subsequent microbiome repopulation from a healthy human donor restored the gut bacteriome diversity typical of a healthy human donor with no significant systemic inflammation.

### 2.2. Effects of Representative GW Chemical Treatment on the Gut Bacteriome, Resistome Profiles, and Systemic Inflammation in CD34+ hu-NSG Mice with Established Human Gut Bacteriome

**Figure 4:**
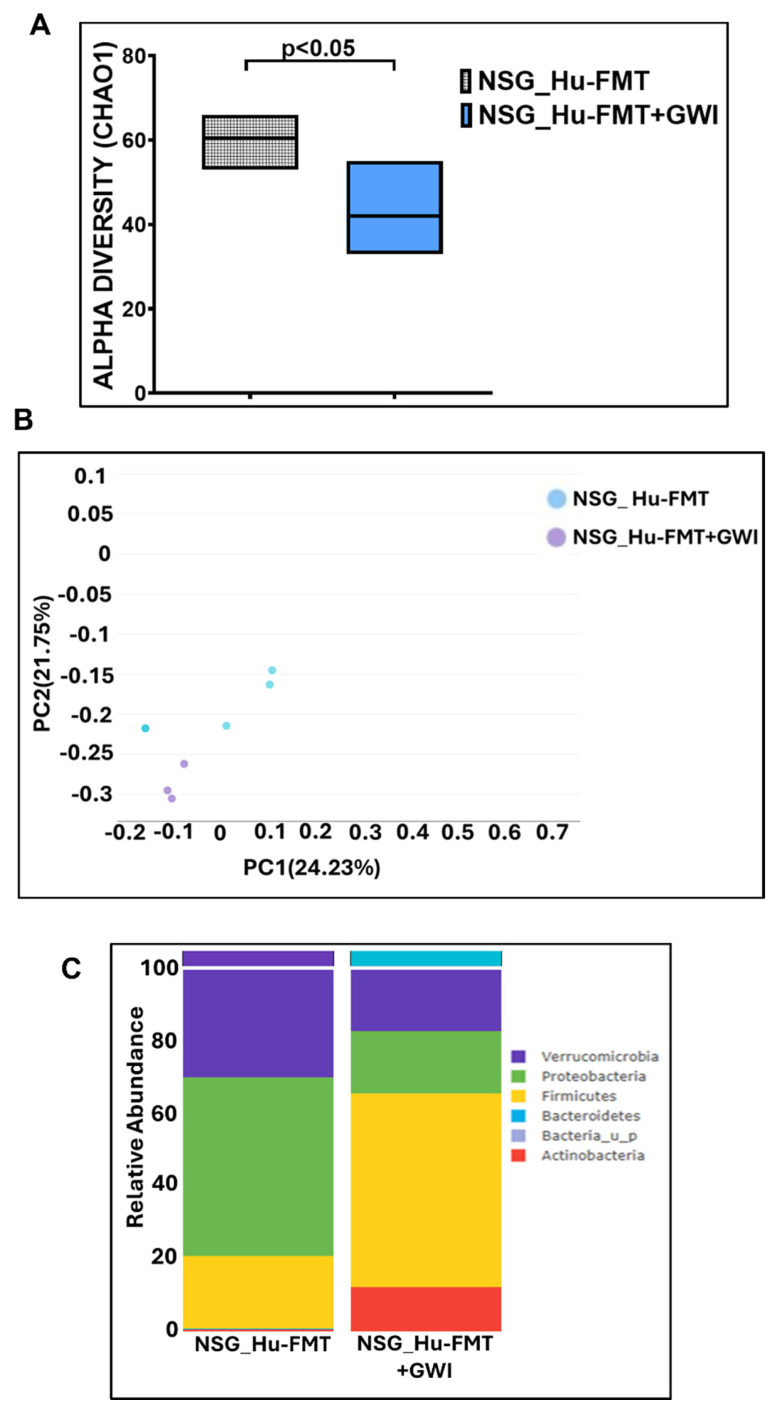
Exposure to representative GW chemicals altered gut bacteriome profile in NSG-CD34+ mice with established human gut bacteria. (**A**) Box plots showing α-diversities of gut bacteriome (Chao 1) in NSG_Hu-FMT (mice administered with human fecal microbiota transfer after gut bacteriome depletion with antibiotic cocktail) and NSG_Hu-FMT+GWI (mice administered with representative GW chemicals pyridostigmine bromide and permethrin for 15 days after gut bacteriome depletion with antibiotic cocktail and human fecal microbiota transfer). (**B**) β-Diversity analysis (Bray–Curtis) of NSG_Hu-FMT and NSG_Hu-FMT+GWI groups. (**C**) Stacked bar representation of relative abundance of gut bacteriome at the phylum level in NSG_Hu-FMT and NSG_Hu-FMT+GWI groups; *p* < 0.05 was considered as statistically significant.

**Figure 5:**
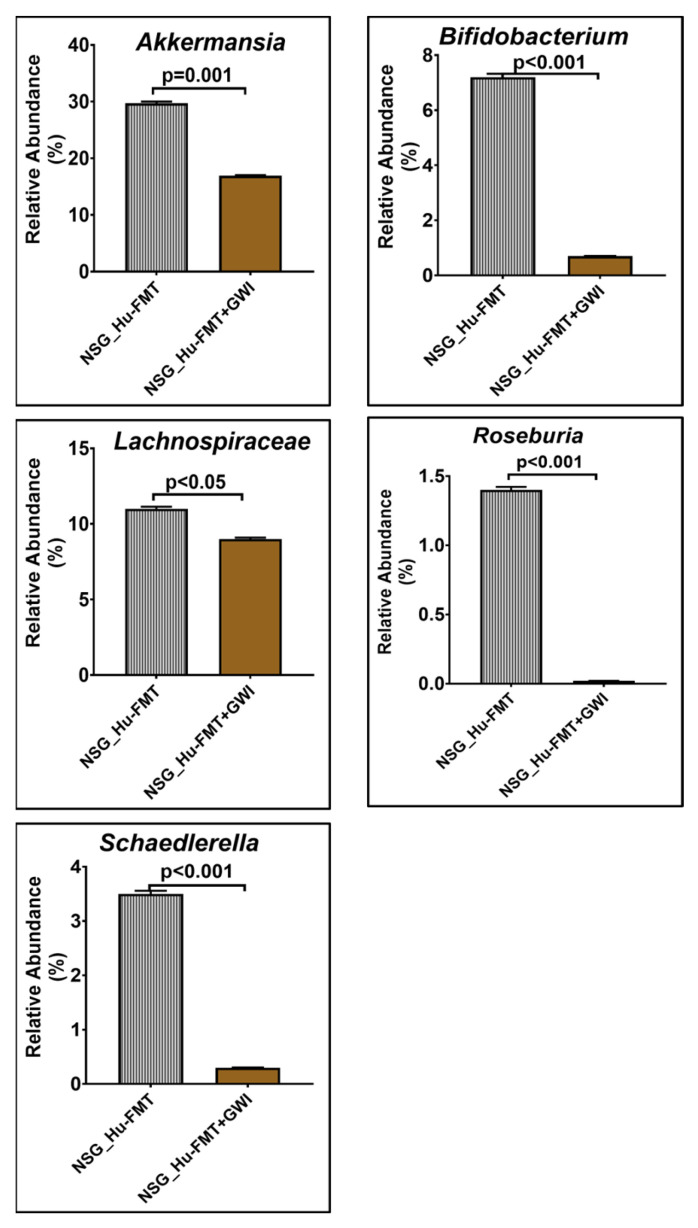
Bar graph representation of relative abundances of gut bacteriome at the genus level in NSG_Hu-FMT and NSG_Hu-FMT+GWI groups; *p* < 0.05 was considered as statistically significant.

**Figure 6:**
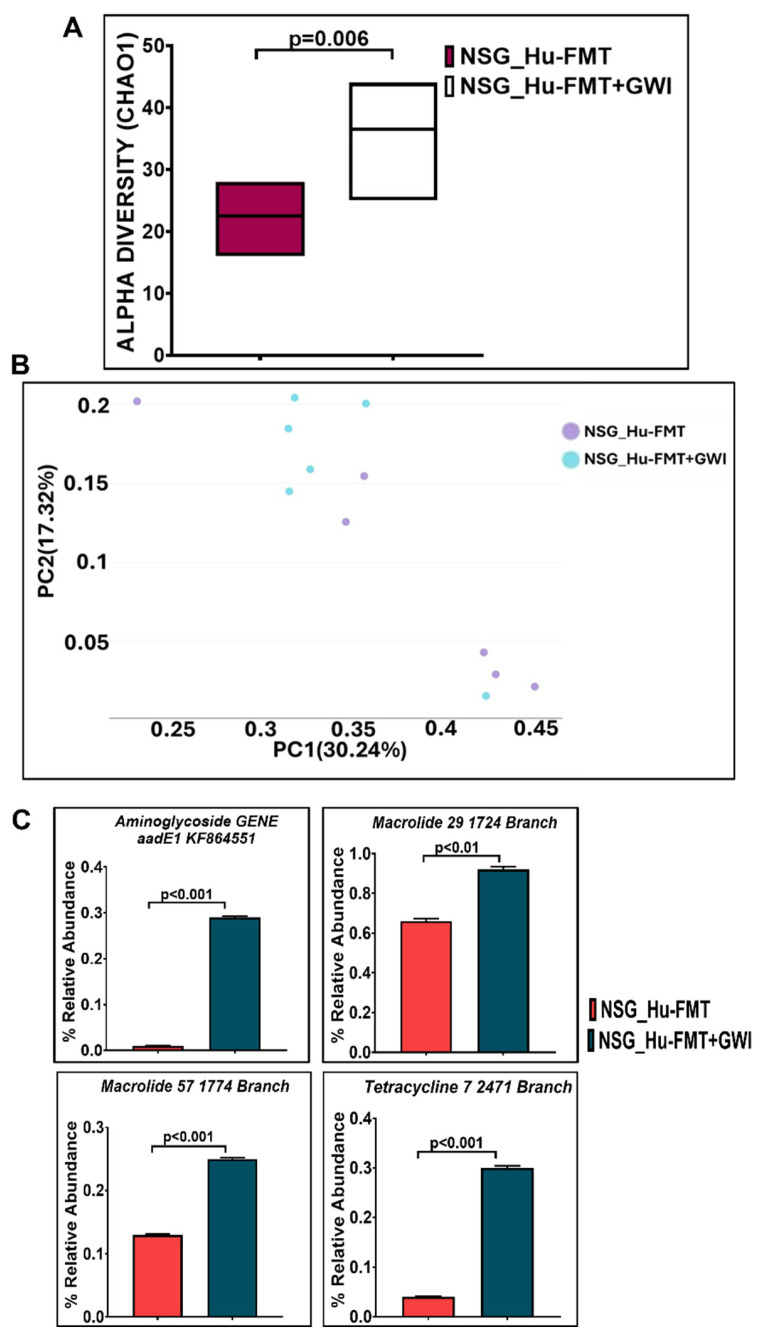
Exposure to representative GW chemicals altered gut resistome profile in NSG-CD34+ mice with established human gut bacteria. (**A**) Box plots showing α-diversities (Chao 1) of gut resistome in NSG_Hu-FMT (mice administered with human fecal microbiota transfer after gut bacteriome depletion with antibiotic cocktail) and NSG_Hu-FMT+GWI (mice administered with representative GW chemicals pyridostigmine bromide and permethrin for 15 days after gut bacteriome depletion with antibiotic cocktail and human fecal microbiota transfer). (**B**) β-Diversity analysis (Bray–Curtis) of NSG_Hu-FMT and NSG_Hu-FMT+GWI groups. (**C**) Bar graph representation of relative abundances of altered antibiotic resistance genes in NSG_Hu-FMT and NSG_Hu-FMT+GWI groups; *p* < 0.05 was considered as statistically significant.

**Figure 7:**
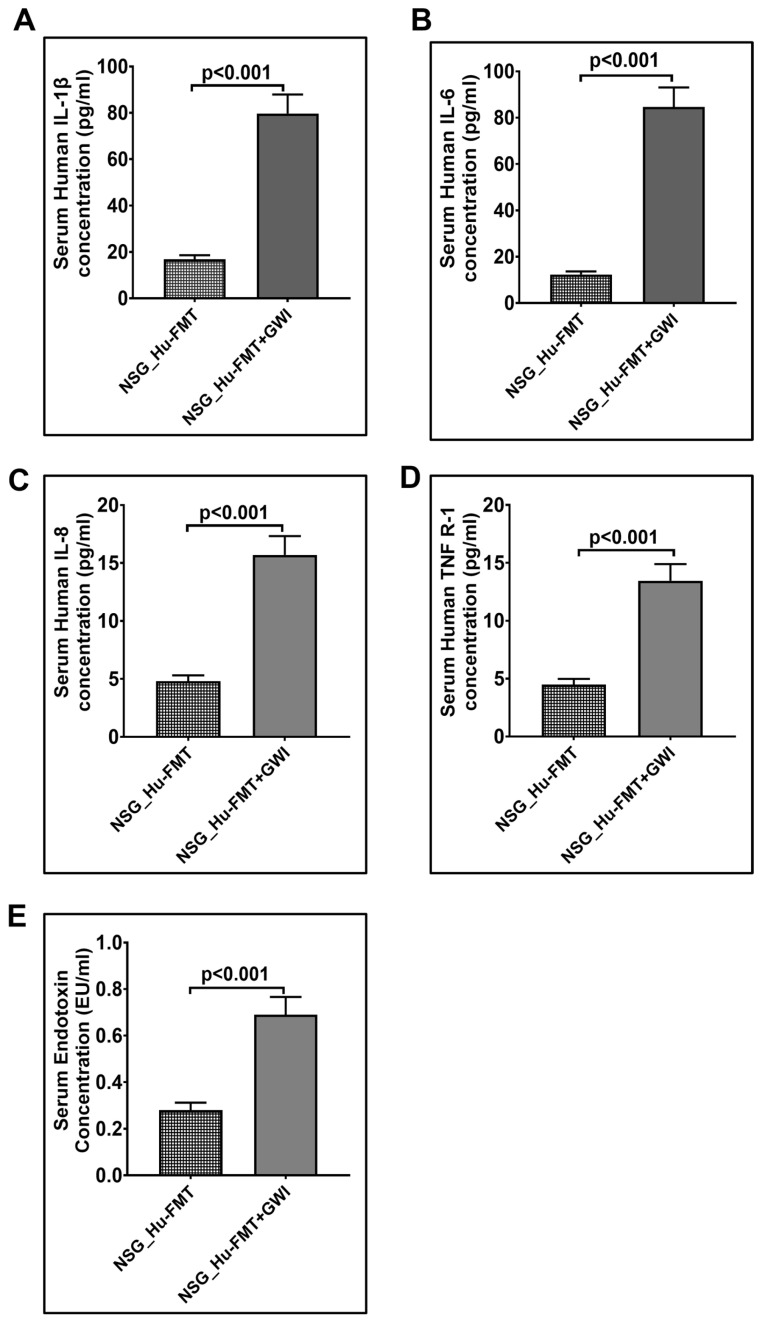
Altered expression of systemic proinflammation biomarkers after administration with representative GW chemicals in NSG-CD34+ mice with established human gut bacteria. Bar graph representation of systemic cytokines (**A**) IL-1β, (**B**) IL-6, (**C**) IL-8, and (**D**) TNF R-1 in NSG_Hu-FMT and NSG_Hu-FMT+GWI groups. (**E**) Bar graph representation of serum endotoxemia, measured by LAL assay in NSG_Hu-FMT and NSG_Hu-FMT+GWI groups; *p* < 0.05 was considered as statistically significant.

The current aim for the below-reported experiments was to use the humanized gut bacteriome model to study the possible alterations in the gut microbial diversity following GW chemical exposure. We studied the alterations in the gut bacteriome and resistome profile after the administration of representative GW chemicals pyridostigmine bromide and permethrin in CD34+ hu-NSG mice having an established human gut bacteriome. The α-diversity (Chao 1) was significantly decreased in the NSG_Hu-FMT+GWI group when compared to the NSG_Hu-FMT group (*p* < 0.05) (Figure 4A). The Bray–Curtis β-diversity analysis, a representation of the richness and unique niches between groups, showed that the observed difference in the gut bacteriome profile between the NSG_Hu-FMT and NSG_Hu-FMT+GWI groups was non-significant (*p* = 0.131) (Figure 4B). At the phylum level, we observed an increase in the relative abundance of *Firmicutes* and a decrease in *Verrucomicrobia* in NSG_Hu-FMT+GWI compared to the NSG_Hu-FMT group (Figure 4C). At genus level, significant decreases were observed in the relative abundances of *Akkermansia* (*p* = 0.001), *Bifidobacterium* (*p* < 0.001), *Lachnospiraceae* (*p* < 0.05), *Roseburia* (*p* < 0.001), and *Schaedlerella* (*p* < 0.001) in the NSG_Hu-FMT+GWI group compared to the NSG_Hu-FMT group (Figure 5). Further, we studied the gut resistome profile between these two experimental mouse groups because our previous study reported the association of the gut resistome alteration with the underlying GWI condition [22]. A significant increase in the α-diversity of antibiotic resistance genes was observed in NSG_Hu-FMT+GWI compared to the NSG_Hu-FMT group (*p* = 0.006) (Figure 6A). The Bray–Curtis β-diversity analysis showed that although there was a distinct separation in the gut resistome profile between the two groups, the difference was statistically non-significant (*p* = 0.141) (Figure 6B). We also observed significant increases in the relative abundances of antibiotic resistance genes *aminoglycoside gene aadE 1 KF864551* (*p* < 0.001), *macrolide 29 1724 Branch* (*p* < 0.01), *macrolide 57 1774 Branch* (*p* < 0.001), and *tetracycline 7 2471 Branch* (*p* < 0.001) in NSG_Hu-FMT+GWI compared to the NSG_Hu-FMT group (Figure 6C). Further, we observed significant increases in the expressions of serum proinflammatory markers IL-1β (*p* < 0.001), IL-6 (*p* < 0.001), and IL-8 (*p* < 0.001) and the soluble TNF R-1 (*p* < 0.001) in NSG_Hu-FMT+GWI compared to the NSG_Hu-FMT group (Figure 7A–D). Further, we observed a significant increase in the endotoxin levels (*p* < 0.001) in NSG_Hu-FMT+GWI compared to the NSG_Hu-FMT group (Figure 7E), suggesting a distinct gut-leaching phenomenon often associated with gut dysbiosis in GWI and other metabolic and environment-linked disease phenotypes.

### 2.3. Effects of Fecal Microbiota Transfer from GWI Veteran’s Stool Sample on the Gut Bacteriome, Resistome Profiles, and Systemic Inflammation in CD34+ hu-NSG Mice

**Figure 8:**
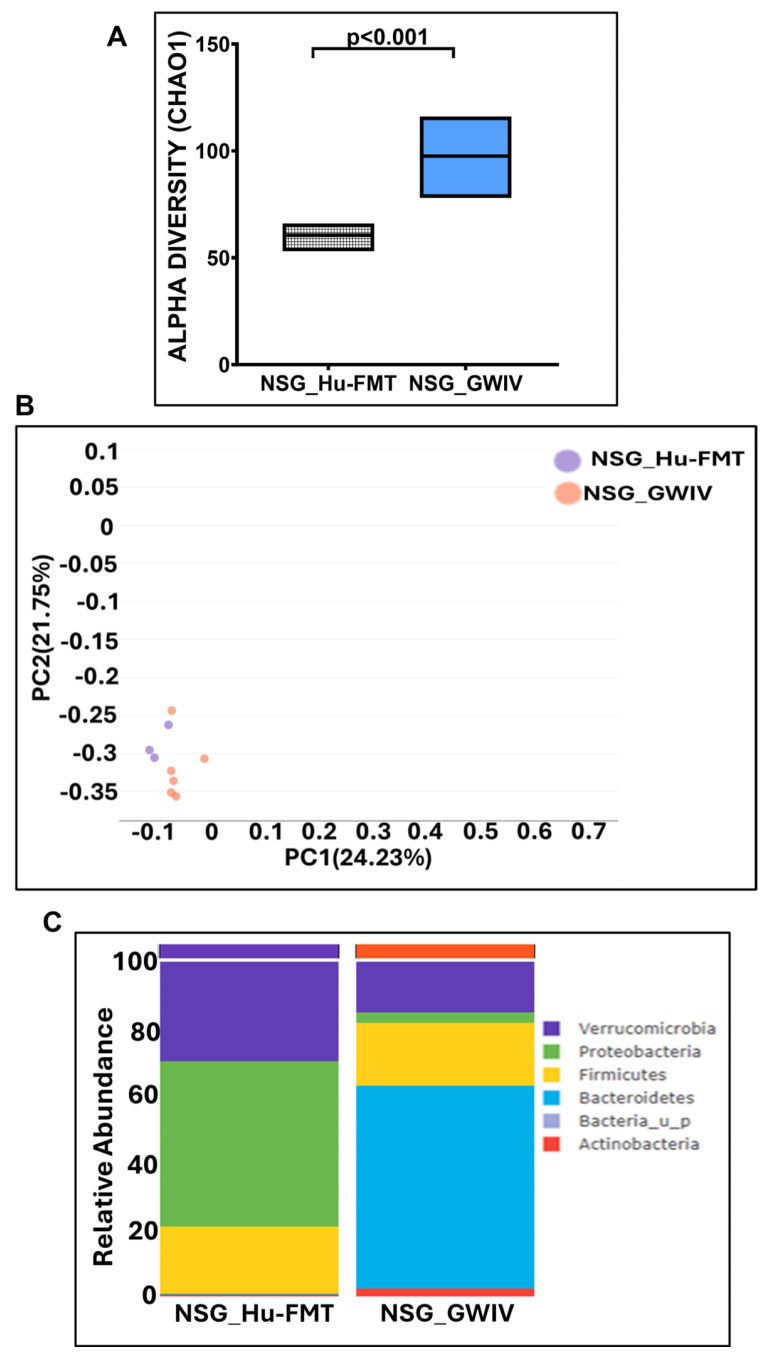
Administration with human fecal microbiota transfer from GWI Veteran’s stool sample altered gut bacteriome profile in NSG-CD34+ mice. (**A**) Box plots showing α-diversities of gut bacteriome (Chao 1) in NSG_Hu-FMT (mice administered with human fecal microbiota transfer after gut bacteriome depletion with antibiotic cocktail) and NSG_GWIV (mice administered with human fecal microbiota transfer from GWI Veteran’s stool sample after gut bacteriome depletion with antibiotic cocktail). (**B**) β-Diversity analysis (Bray–Curtis) of NSG_Hu-FMT and NSG_GWIV groups. (**C**) Stacked bar representation of relative abundances of gut bacteriome at the phylum level in NSG_Hu-FMT and NSG_GWIV groups; *p* < 0.05 was considered as statistically significant.

**Figure 9:**
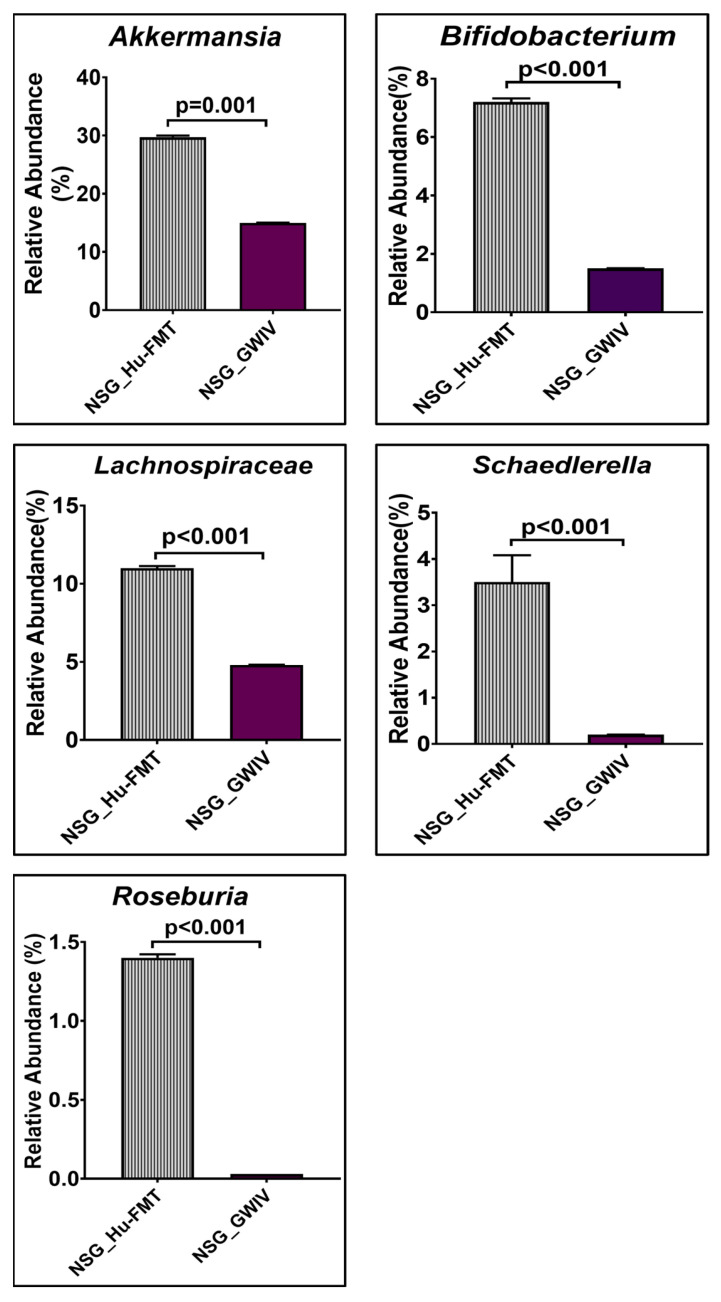
Bar graph representation of relative abundances of gut bacteriome at the genus level in NSG_Hu-FMT and NSG_GWIV groups; *p* < 0.05 was considered as statistically significant.

**Figure 10:**
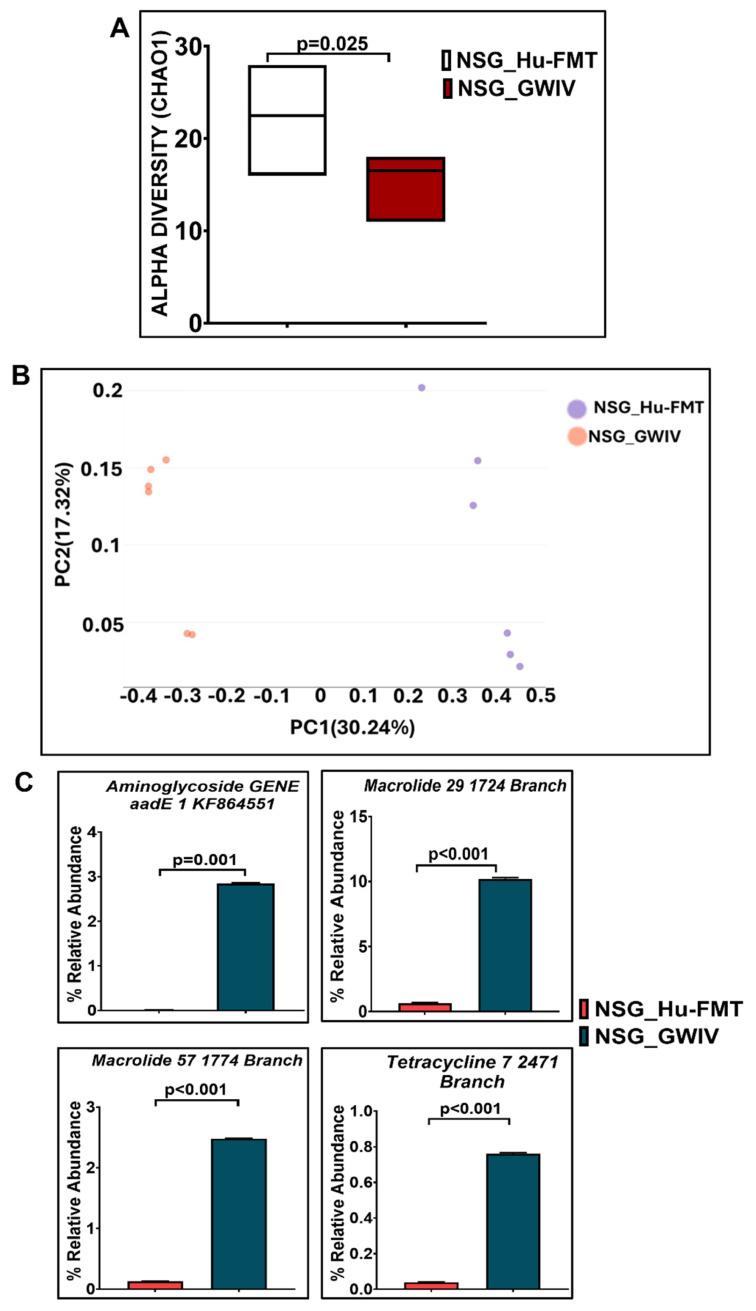
Administration with human fecal microbiota transfer from GWI Veteran’s stool sample altered gut resistome profile in NSG-CD34+ mice. (**A**) Box plots showing α-diversities (Chao 1) of gut resistome in NSG_Hu-FMT (mice administered with human fecal microbiota transfer after gut bacteriome depletion with antibiotic cocktail) and NSG_GWIV (mice administered with human fecal microbiota transfer from GWI Veteran’s stool sample after gut bacteriome depletion with antibiotic cocktail. (**B**) β-Diversity analysis (Bray–Curtis) of NSG_Hu-FMT and NSG_GWIV groups. (**C**) Bar graph representation of relative abundances of altered antibiotic resistance genes in NSG_Hu-FMT and NSG_GWIV groups; *p* < 0.05 was considered as statistically significant.

**Figure 11:**
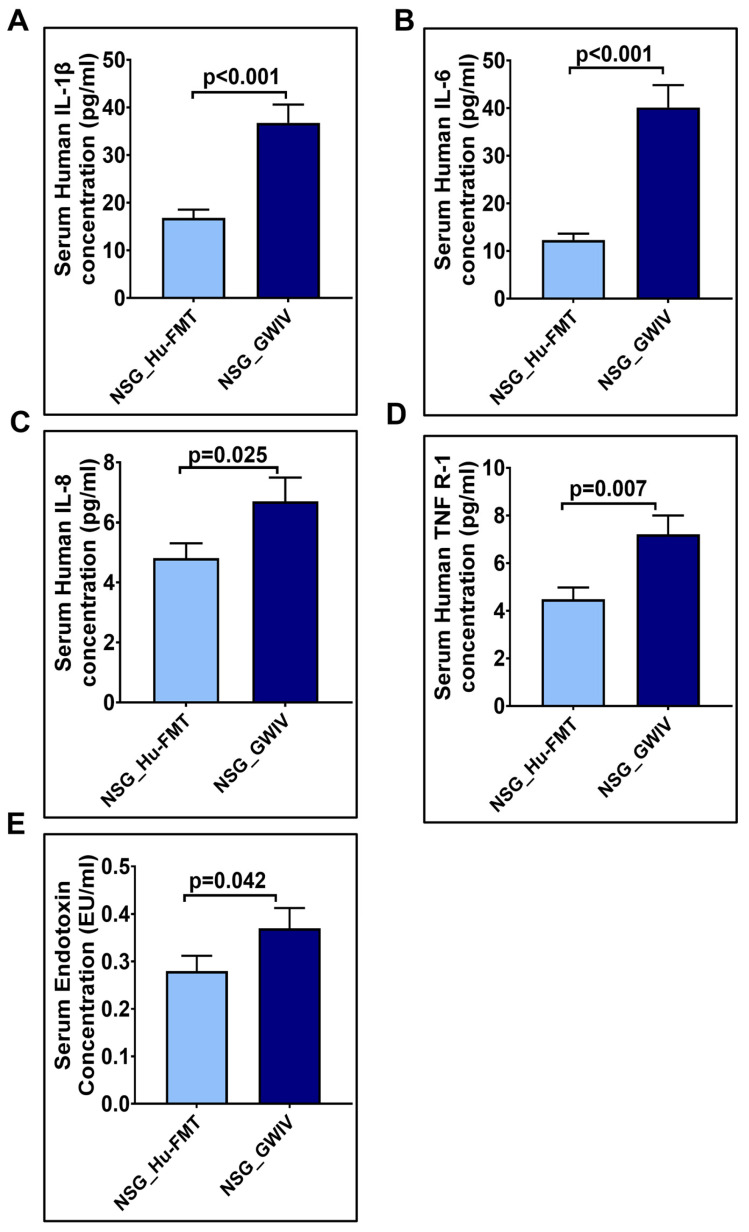
Altered expressions of systemic proinflammation biomarkers, after human fecal microbiota transfer from GWI Veteran’s stool sample, in NSG-CD34+ mice. Bar graph representation of systemic cytokines (**A**) IL-1β, (**B**) IL-6, (**C**) IL-8, and (**D**) TNF R-1 in NSG_Hu-FMT and NSG_GWIV groups. (**E**) Bar graph representation of serum endotoxemia, measured by LAL assay in NSG_Hu-FMT and NSG_GWIV groups; *p* < 0.05 was considered as statistically significant.

Further, we wanted to study whether establishing the gut bacteriome from GWI Veterans by FMT would result in similar alterations in the gut bacteriome, resistome, and systemic inflammatory patterns in the new GWI-mouse model discussed in the previous section. The aim was to recreate a unique gut microbiome specific to a deployed GWI Veteran. We compared the results of this NSG_GWIV group (humanized mouse group with GWI Veteran microbiome) with the results of the NSG_Hu-FMT group, which mimics the health condition of a healthy human supposedly representing a non-deployed individual. The results showed that the α-diversity (Chao 1) of the NSG_GWIV group was significantly increased (*p* < 0.001) compared to the NSG_Hu-FMT group (Figure 8A). We observed that the gut bacteriome profiles of the two groups were significantly distinct (*p* = 0.004) from each other by the Bray–Curtis β-diversity analysis (Figure 8B). At the phylum level, the relative abundance of *Verrucomicrobia* was decreased in the NSG_GWIV group compared to the NSG_Hu-FMT group (Figure 8C). However, no change was observed in the relative abundance of *Firmicutes* (Figure 8C). Further, we compared the gut bacterial profile at the genus level in the GWI Veteran’s fecal sample (Supplementary Materials Table S1) with the established bacterial profile in the NSG_GWIV group and observed the following changes: At the genus level, we observed significant decreases in the relative abundances of *Akkermansia* (*p* = 0.001), *Bifidobacterium* (*p* < 0.001), *Lachnospiraceae* (*p* < 0.001), *Roseburia* (*p* < 0.001), and *Schaedlerella* (*p* < 0.001) in the NSG_GWIV group compared to the NSG_Hu-FMT group (Figure 9). We also observed that the α-diversity (Chao 1) of the antibiotic resistance gene profile in the NSG_GWIV group was significantly altered as compared to that in the NSG_Hu-FMT group (*p* = 0.025) (Figure 10A). The Bray–Curtis β-diversity analysis showed a significant difference (*p* < 0.003) between the gut resistome profiles of the two groups (Figure 10B). At the individual gene level, we observed significant increases in the relative abundances of *aminoglycoside gene aadE 1 KF864551* (*p* = 0.001), *macrolide 29 1724 Branch* (*p* < 0.001), *macrolide 57 1774 Branch* (*p* < 0.001), and *tetracycline 7 2471 Branch* (*p* < 0.001) in NSG_GWIV compared to the NSG_Hu-FMT group (Figure 10C). Finally, we studied whether the colonized bacteriome from the GWI Veteran resulted in the expression of systemic proinflammatory biomarkers. Significant increases in the expressions of IL-1β (*p* < 0.001), IL-6 (*p* < 0.001), and IL-8 (*p* = 0.025) and the soluble TNF R-1 (*p* = 0.007) were observed in the NSG_GWIV group compared to the NSG_Hu-FMT group (Figure 11A–D). A significant increase in the endotoxin levels (*p* = 0.042) in NSG_GWIV was observed when compared to the NSG_Hu-FMT group (Figure 11E), suggesting that our novel approach to recreate the GWI microbiome in a humanized-mouse model bore similarities with the average GWI Veteran having systemic inflammation, as reported in several human studies [23,24].

## 3. Discussion

Our study is the first attempt to recreate a mouse model of GWI that reflects a human immune system and a human gut microbiome. Owing to many previous research studies, primarily conducted on mouse or rat models, several mechanistic pathways related to the pathology of GWI have been identified [3,4,25]. These studies helped us to understand the possible therapeutic targets ranging from systemic inflammation to neuroinflammation and gut microbiome alterations. However, many such studies differed in the endpoints when related to human cohorts and patient outcomes, though the authors agree that various etiologies of GWI, such as a combination of exposures, routes of exposure, and symptom persistence, provided valuable information for their translatability in humans in spite of the limitations for using an animal model. Further, animal models can provide for the high reproducibility of outcomes when imposing conditions as observed in the GW theater and can help to summarize the translational value, especially when identifying the treatments, tolerability, and efficacy of new treatment strategies. For example, our own studies in mouse models that reported gut bacteriome alterations showed marked differences in cytokine profiles and microbiome diversity when compared to a pilot study in humans [14,17]. It is not unknown that mouse microbiome profiles are distinctly different when compared to human microbiota, and it is difficult to correlate the mouse pathology arising from such a diverse microbiome to a possible human disease interface [26]. Further, mouse gut-associated lymphoid tissue (GALT) and its interactions with the specific mouse immune phenotype may have subtle differences with a human immunophenotype [27]. The above challenges have led investigators wishing to study gut microbiome–immune interactions to switch to mouse models that effectively have a human microbiome coupled with a human immune system [28]. Though such a hypothesis is easier stated than proven, the use of novel methodologies and implementation in disease models may have challenges. For example, the use of immunocompromised mice and their resistance or absence for overcoming a series of surgical interventions, as well as exposure to repeated drugs/handling stress can have severe implications on the health of the mice [12]. Moreover, the costs and sophistication involved in conducting such experiments have limited their use. In this study, we have attempted to use an established mouse strain commercially available from Jackson Laboratories (NSG^TM^, engrafted with bone-marrow-derived CD34+) to supplant a human microbiome, following a modified protocol previously used by Daharsh et al. [12]. We also used two distinct approaches to recreate a GWI Veteran phenotype in the mouse. First, we recreated a more established model, widely used in the GWI pathology field, that uses GW chemicals, permethrin and pyridostigmine bromide, as the routine exposure, coupled with the transplantation of a healthy human microbiome to facilitate the human–gut–immune interaction, and another novel concept to repopulate the GWI Veteran’s microbiome in the mice that already had a human immunophenotype.

The procedure to create a double humanized engraftment comes with numerous challenges. The one that had to be dealt with relied on the successful engraftment of a healthy human microbiome after the depletion of a mouse microbiome. The results showed that the mouse microbiome deletion with a cocktail of antibiotics significantly decreased the α-diversity of the host’s gut microbiota but was restored to a higher diversity upon human microbiome engraftment (Figure 1A). Notably, our whole-genome sequencing approach allowed us to dive deep into the species diversity, and the results showed that healthy human microbiome engraftment significantly restored several probiotic species, which were otherwise decreased upon antibiotic treatment (Figure 2). The above approach and data confirmed that though complex, we successfully repopulated the human microbiota after depleting the mouse microbiota in these mice. Further, the increased systemic inflammation that followed the antibiotic treatment, a natural occurrence due to the severe stress involved, was also restored to normal levels upon the human healthy microbiota engraftment, another evidence that the mice recovered fully after the human microbiota was engrafted. There was no residual inflammation that could skew our results if and when these same mice were to be exposed to disease mechanisms (Figure 3).

Having successfully engrafted the human microbiota, we tested whether the gut–immune axis in a double humanized microenvironment withstood the test of the GW chemical exposure and bore a resemblance to the present-day GWI Veteran. The results showed that GW chemical exposure showed an altered microbiota and an increased systemic inflammatory profile in this model when compared to non-exposed mice (Figure 4, Figure 5, Figure 6 and Figure 7). Notably, the resistome profile also showed an altered signature similar to those of human cohorts from our previously published study [14]. In this study, the increased expression of antibiotic resistance genes in the NSG_Hu-FMT+GWI group could result from selection pressure on the gut microbiome created because of the administration of GW chemicals. Studies have reported that increased exposure to environmental chemicals results in increased expression of these genes [29]. This could also be due to horizontal transfer via mobile genetic elements that are harbored by the gut bacteria bearing these resistance genes [30]. Tetracycline and macrolide are among the commonly prescribed antibiotics in clinics and hospitals; hence, resistance to these antibiotics might lead to treatment failures in aging GWI Veterans in the future [31]. We should emphasize, however, that the β-diversity in the GW-chemical-exposed mice had a non-significant difference when compared to the humanized controls. Still, this result opened a new avenue to explore a deeper approach for studying the individual species abundance not explored in human exposure. The results showed that the probiotic bacteria and principal short chain fatty acid (SCFA)-producing bacteria were significantly decreased in the GW-chemical-exposed humanized mice when compared to controls [32]. The results also confirmed the need to use SCFA supplementation as an effective therapeutic modality as the one being tested now by our group [33]. Notably, a previous pilot study from our group found a significant increase in TNF R-1 levels in GWI Veterans, a result that was similar to that in our humanized-mouse model, thus confirming that the present model may be a feasible alternative to a murine drug discovery platform confined for using only the mouse immune system and/or mouse immune phenotype [14].

There are various limitations in using a GW-chemical-induced model for studying pathology, and though several mouse models have been used to study the symptoms of GWI, no one model represents a true exposome of GWI. Often, studies on mouse models do not reflect the true exposure of either chemicals or mixtures that may have been present in the war theater. For example, GWI is the result of a mixture of exposures, such as organophosphate insecticides, oil well smoke, and consumption of pyridostigmine bromide ( pills. Many studies have focused only on pyridostigmine bromide as a model exposure, severely limiting the thorough study of the GWI exposome. The above exposure paradigms cause several variabilities in data interpretation, as we do not know the proportion of each exposure component in an ideal physiological and pharmacological setting. We, therefore, used another novel approach (apart from a mixture of GW chemicals) to mimic exposure or recreate a true GW exposome using microbiome engraftment as an option. As found in our earlier pilot study, the above hypothesis originated with the assumption that gut microbiome dysbiosis is a key predictor of many GWI symptoms, including gastrointestinal disturbances and systemic immune alterations. In this aim, we engrafted the human microbiota derived from a deployed GWI Veteran who had persistent GWI with most of the prominent symptoms, as noted from a list of Veterans with GWI from the George E. Wahlen Veterans Affairs Medical Center’s (GEWVAMC’s) Gulf War registry [34]. A single Veteran’s stool sample was chosen to be a representative of a GWI cohort, where gut dysbiosis and gastrointestinal disturbances coexist. A pooled stool sample from all the GWI Veterans’ stool samples was considered but was not feasible, as that approach would generalize the disease phenotype. A future study may consider transplanting bacterial colonies from each Veteran’s stool sample to each subsequent mouse to represent each individual in the cohort. The control group received a microbiota transplant from a healthy non-deployed volunteer. The results showed that the engrafted human microbiota group from the deployed individual had significantly decreased probiotic species abundance similar to what was found in the GW-chemical-exposure group (Figure 8, Figure 9 and Figure 10). This group also had a similar resistome profile and significant elevations in TNF R-1, IL-1β, and IL-6, a profile that matched our earlier pilot study with the GWI human cohort (Figure 11).

In this study, several major breakthroughs were achieved. This study is the first to create a model that closely resembles a GWI exposome, albeit mimicking the human exposure and its immune phenotype more closely than a typical mouse model. Second, this study considers a more controlled human microbiota behavior and its interactions with the immune system, as more studies confirm the important role of the host’s gut microbiome in the pathology of multisymptom illnesses. This model can be used for various exposures more often associated with GWI, such as oil well smoke and side effects of vaccines, apart from the regular and more common exposures, such as insecticides and pyridostigmine bromide. Further, the present model can be used in studying more systemic pathologies, such as chronic fatigue, as fatigue is known to influence gut microbiome–immune system interactions. Interestingly, our model can also play a broader role in pathological studies involving chronic fatigue syndrome. For example, we know that chronic fatigue can result from mitochondrial abnormalities, the shortening of telomeres, redox stress, and, the most importantly, irregularities in the immune system [35]. Immune system disorders can result from immunosenescence, a condition where the immune system can become exhausted, chronic low-inflammatory triggers arise because of consistently higher levels of IL-6, and T-lymphocyte subsets are abnormally activated [36]. The present human immune system reconstitution model can help to identify immune system abnormalities, predict immune cell senescence, and even study mitochondrial abnormalities in these cells. Further, our present study can be of immense predictive and translational value, considering the challenges we face in identifying treatment strategies in GWI owing to its complex etiology. For example, feasible small-molecule drug interventions, which may be endogenous metabolites or a probiotic, can be tested in this model for possible safety, tolerability, and positive outcomes before initiating a Phase I or Phase IIa trial. Though the results of our study provide a feasible alternative for existing pathology and drug discovery models in GWI, we have identified several limitations and alternative approaches that need to be resolved.

Though NSG-CD34+ mice are a viable humanized model for human microbiota engraftment, bone-marrow-, thymus-, and liver-engrafted mice in an NSG background or BLT engraftment in triple knockout (TKO) (CD47−/−RAG1−/−IL15Ra−/−) immunodeficient mice in a C57BL/6 background might have better results because GALT structures are better defined in these models [10]. Further, humanized-mouse models often have severe graft-versus-host disease, which can have either an early or a late onset, depending on the model. NSG mice supplanted with CD34+ cells, as used in our model, may suffer from insufficient engraftment [37,38]. Our model did not have an early onset of graft-versus-host (GVH) disease, but we were severely limited in extending our studies for more than 15 weeks post exposure. Interestingly, many GWI studies using mouse models and chemical exposures used a 20-week resting period before the study termination to prove symptom persistence. In these models, it would be advantageous to use a BLT-TKO-humanized model with a very late GVH onset (>45-week onset).

GWI pathology has been studied related to its manifestation in the brain, especially in areas of neuroinflammation, pain, and cognitive dysfunction [39,40]. We have shown that gut microbiome dysbiosis may play a role in neuroinflammation [15]. A double humanized mouse model, such as that discussed in the present study, may not be suitable for studying organ manifestations distant to the gut, as the brain tissue in these mice is not humanized. This will be true for secondary organ targets, like the liver and kidneys. Another possible limitation is the use of GWI Veterans’ microbiota transfer for recreating/mimicking the typical GWI Veteran. Interestingly, the human microbiome is diverse, and several differences exist between individuals. No one Veteran’s stool sample may be a true representative of a cohort. To ideally represent the GWI cohort in a humanized model, each mouse transplanted with the Veteran’s stool microbiota should represent a single Veteran rather than generalize the entire cohort by pooling the stool samples or using just 2–3 representative microbiome engraftments from stool samples. This process may be extensively rigorous, but engrafting and recreating in a mouse a humanized microbiome from each individual Veteran will create a true cohort accurately representing each Veteran, as is conducted in a clinical study. The above approach and a humanized immune system similar to what has been described in this study may serve as an ideal humanized model for GWI.

## 4. Materials and Methods

Pyridostigmine bromide, permethrin, metronidazole, neomycin, vancomycin, ampicillin and all other chemicals unless specified were purchased from Sigma-Aldrich (St. Louis, MO, USA).

### 4.1. Animals

We purchased pathogen-free, adult (21 weeks old), female CD34+ hu-NSG mice from Jackson Laboratories (Bar Harbor, ME, USA). Upon arrival, all the mice were accommodated in a room with a controlled temperature (22–24 °C), having a 12 h light/12 h dark cycle and ad libitum access to food and water. The mice were housed under SPF conditions with air exchange, prefilters, and HEPA filters (0.22 μm) in a room with controlled temperature, humidity, and pressure. The mice were maintained in autoclaved individual microisolator cages in a rack system capable for managing air exchange with prefilters and HEPA filters (0.22 μm). All the experiments with the mice were approved by the University of California Irvine and followed the local Institutional Animal Care and Use Committee’s standards (protocol number AUP-23-015, approved on 14 April 2023).

### 4.2. Mouse Model of Gulf War Illness

The mice were acclimatized for one week, followed by random distribution into five experimental groups, where each group comprised 6 mice. The NSG_Control mouse group was administered with a phosphate-buffered saline solution via oral gavage for 30 days. The NSG_ABX Treatment group was administered with an antibiotic cocktail solution (metronidazole, 1 g/kg; neomycin, 1 g/kg; vancomycin, 0.5 g/kg; and ampicillin, 1 g/kg) via oral gavage for 12 days and allowed to persist for the remaining 18-day period. The NSG_Hu-FMT group was administered with the antibiotic cocktail via oral gavage for the initial 12 days for the native gut bacteriome depletion, after which FMT via oral gavage was performed for the next 2 days to establish the gut bacteriome of a healthy human. Healthy human fecal samples were purchased from Creative Biomart, Inc. (Shirley, NY, USA), dissolved in sterile phosphate-buffered saline solution, and centrifuged at 3000× *g* for 5 min. Then, 100 µL of the supernatant was administered in the mice. After the FMT procedure, the mice were allowed to persist for the remaining 15 days. It is immensely important that correct procedures be followed for FMT, including the volume, medium, and number of bacteria transplanted [41]. For the present study, we used 10^8^ CFU per/mL [42]. In the NSG_Hu-FMT+GWI group, gut depletion was conducted by antibiotic cocktail administration via oral gavage for 12 days, and human bacteriome establishment was conducted by performing FMT similar to that in the NSG_Hu-FMT group. After the FMT process, representative GW chemicals, pyridostigmine bromide (2 mg/kg diluted in phosphate-buffered saline) and permethrin (200 mg/kg diluted in 0.6% dimethyl sulfoxide), were administered via oral gavage on a triweekly basis for 15 days. The NSG_GWIV mouse group was administered with an antibiotic cocktail via oral gavage for 12 days for gut bacteriome depletion followed by an FMT process with a GWI Veteran’s stool sample for 2 days. The stool sample was homogenized in 1 mL of phosphate-buffered saline solution, and centrifugation was performed at 3000× *g* for 5 min. Then, 100 µL of the supernatant was immediately administered in the NSG_GWIV mouse group. The deidentified GWI Veterans’ stool samples were kindly provided by Dr. Tuteja from his recently conducted study [34]. The human cohort study from which the human stool sample was collected was approved by the Salt Lake City Veterans Affairs Medical Center and the University of Utah Institutional Review Board. The study was registered at ClinicalTrials.gov (NCT03078530). These mice were allowed to persist for the remaining 15 days for the effective repopulation of the gut bacteriome. All the mice were sacrificed after the experimental period, fecal pellets were collected for the bacteriome analysis, and serum was collected from blood freshly collected by the process of cardiac puncture.

### 4.3. Bacteriome Analysis

Fecal pellets were collected from all the experimental mouse groups for bacteriome analysis by the vendor CosmosID, Inc. (Germantown, MD, USA). Briefly, the total DNA from the fecal pellets was isolated following the manufacturer’s protocol using QIAGEN DNeasy PowerSoil Pro Kit (Germantown, MD, USA). The quantification of the extracted DNA was performed using a Qubit Flex fluorometer and a Qubit dsDNA HS assay kit (Thermo Fisher Scientific, Waltham, MA, USA). The DNA library was prepared using the xGen DNA library prep kit (IDT, Coralville, IA, USA) and xGen Normalase UDI primers, with a total DNA input of 1.5 ng. DNA fragmentation was performed using a specified amount of the IDT xGEN fragmentation enzyme (Coralville, IA, USA). The library was constructed by adding unique dual indices to each sample, followed by 10 cycles of PCR. The DNA libraries were purified using AMpure magnetic beads (Beckman Coulter, Indianapolis, IA, USA) and eluted in QIAGEN EB buffer (Germantown, MD, USA). The DNA libraries were quantified using a Qubit fluorometer and a Qubit dsDNA HS assay kit. The libraries were sequenced on the Illumina NovaSeq X Plus (San Diego, CA, USA) platform at 2 × 150 bp. The data, upon arrival, were run through fastqc (version 0.11.9). The multiqc report was reviewed to ensure that the read depths met the thresholds and there were no errors with duplication rates, read quality, and adaptor content. The results for the taxonomic analysis were viewed on the vendor’s CosmosID-Hub microbiome platform to ensure there were no barcoding or contamination issues.

### 4.4. Enzyme-Linked Immunosorbent Assay (ELISA)

ELISA was performed using serum collected from the mice using commercially available kits for human IL-1β (Proteintech, Rosemont, IL, USA), human IL-6 (Proteintech, Rosemont, IL, USA), human IL-8 (Proteintech, Rosemont, IL, USA), and human TNF R-1 (R&D Systems, Inc., Minneapolis, MN, USA). The procedure was carried out following the manufacturer’s protocol.

### 4.5. Limulus Lysate Test (LAL) Assay

The serum endotoxin levels were quantified using a Pierce LAL chromogenic endotoxin quantitation kit from Thermo Fisher Scientific (Waltham, MA, USA). The procedure was carried out following the manufacturer’s protocol.

### 4.6. Statistical Analysis

The statistical analyses used GraphPad Prism software version 10.2.2 (San Diego, CA, USA). One-way ANOVA and unpaired two-tailed *t*-tests were conducted with Bonferroni–Dunn post hoc corrections. The data are represented as means ± SEM; *p* ≤ 0.05 was considered to be statistically significant for all the analyses.

## Data Availability

The data presented in this study are available on request from the corresponding author.

## Supplementary Materials

The supporting information can be downloaded at: https://www.mdpi.com/article/10.3390/ijms25116093/s1.
