# Supplementary material for: A Double-Humanized Mouse Model for Studying Host Gut Microbiome–Immune Interactions in Gulf War Illness
Source: Int J Mol Sci. 2024 May 31;25(11):6093. doi: 10.3390/ijms25116093 (PMC11172868; doi:10.3390/ijms25116093)
Supplement: Supplementary file 1 [file ijms-25-06093-s001.zip › ijms-3008835-supplementary.pdf]

Table S1. Composition of donor gut bacteria at Genus level

Composition of healthy human donor gut bacteria at Genus level	Composition of Gulf War Veteran gut bacteria at Genus level
Bifidobacterium	*Bifidobacterium*
Lachnospiraceae	*Roseburia*
Roseburia	*Akkermansia*
Bacteroides	*Lachnospiraceae*
Lactobacillus	*Schaedlerella*
Eubacterium	*Blautia*
Ruminococcus	*Faecalibacterium*
Coprococcus	*Bacteroides*
Dorea	*Streptococcus*
Faecalibacterium	*Anaerostipes*
Clostridium	*Dorea*
Parasutterella	*Ruminococcus*
Phascolarctobacterium	*Intestinibacter*
Gemmiger	*Coprococcus*
Lachnoclostridium	*Parabacteroides*
	Butyricicoccus
	Alistipes
	Lachnoclostridium
	Turicibacter
	Eggerthella
